# Transient metabolic improvement in obese mice treated with navitoclax or dasatinib/quercetin

**DOI:** 10.18632/aging.103607

**Published:** 2020-06-25

**Authors:** Arantzazu Sierra-Ramirez, José Luis López-Aceituno, Luis Filipe Costa-Machado, Adrián Plaza, Marta Barradas, Pablo Jose Fernandez-Marcos

**Affiliations:** 1 Metabolic Syndrome Group – BIOPROMET, Madrid Institute for Advanced Studies - IMDEA Food, CEI UAM+CSIC, Madrid, Spain

**Keywords:** senescence, obesity, senolytic, diabetes, mouse model

## Abstract

Senescent cells accumulate with obesity in the white adipose tissue of mice and humans. These senescent cells enhance the pro-inflammatory environment that, with time, contributes to the onset of glucose intolerance and type 2 diabetes. Glucose intolerance in mouse models of obesity has been successfully reversed by the elimination of senescent cells with the senolytic compounds navitoclax or the combination of dasatinib and quercetin (D/Q). In this work, we generated obese mice by high-fat diet feeding, and treated them with five consecutive cycles of navitoclax or D/Q during 16 weeks. We observed an efficient reduction in the white adipose tissue of the senescence markers senescence-associated β-galactosidase activity, *Cdkn2a-p16* and *Cdkn2a-p19* at the end of the 5 cycles. Mice treated with both navitoclax and D/Q showed an improvement of their insulin sensitivity and glucose tolerance during a short period of time (cycles 3 and 4), that disappeared at the fifth cycle. Also, these mice tended to increase the expression at their adipose tissue of the adipogenic genes *Pparg* and, *Cebpa*, as well as their plasma adiponectin levels. Together, our work shows that two different senolytic treatments, acting through independent pathways, are transiently effective in the treatment of obesity-induced metabolic disorders.

## INTRODUCTION

Obesity has become a worldwide health problem, with an incidence that has nearly tripled since 1975 [1]. It is defined as an imbalance between energy intake and expenditure that, when maintained in time, causes a systemic chronic inflammation that degenerates in obesity-associated insulin resistance and type 2 diabetes [2].

Previous studies have shown that one of the mechanisms by which chronic obesity leads to type 2 diabetes is the onset of senescence in certain tissues, such as white adipose or pancreatic β-cells [3–5]. Senescence was first described by Hayflick and Moorhead as an *in vitro* limited replicative capacity of normal human fibroblasts [6]. Since then, the definition of senescence has evolved significantly, and it is now generally accepted that senescence is a stable proliferative arrest caused by some stresses such as telomere dysfunction, oxidative stress, DNA damage, oncogene activation or cytotoxic drugs [7, 8]. The pathways induced by these stimuli finally converge in a set of senescence genes, the best known of which are the tumor suppressors *Cdkn2a-p16*, *Cdkn2a-p19*, *Cdkn1b-p27*, *Cdkn2b-p15* or *Cdkn1a-p21*, resulting in proliferative arrest [9, 10]. The most frequently used biomarker to detect senescent cells is the lysosomal β-galactosidase enzyme activity at a suboptimal pH 6.0, known as senescence-associated β-galactosidase staining (SA-βGal) [11].

Senescent cells are metabolically active and induce a complex pro-inflammatory program known as senescence-associated secretory phenotype (SASP) composed by proinflammatory cytokines, chemokines and growth factors [12]. In a healthy scenario, SASP acts in both an autocrine and a paracrine manner to create an inflammatory environment that attracts immune cells to clear senescent cells and promote tissue regeneration [13]. However, when maintained chronically, SASP effects can induce several pathologies such as idiopathic pulmonary fibrosis, cystic fibrosis, lung fibrosis, sarcopenia, cataracts, obesity and type 2 diabetes [14]. In particular, during obesity, accumulation of large amounts of fat in adipocytes triggers an inflammatory response [2]. Also, SA-βGal activity and increased protein expression of the senescence markers p53 and p21 have been reported in adipose tissue from obese humans and mice [3].

Baker and collaborators demonstrated for the first time that removal of senescent cells is beneficial for age-related diseases, using a mouse model that specifically eliminates Cdkn2a-p16-positive cells, named INK-ATTAC [15]. In turn, new compounds were discovered that selectively induced apoptosis in senescence cells, that were termed senolytics [16]. The description of the first senolytic treatments by the combination of dasatinib with quercetin *in vitro* [17], or the inhibitor of the Bcl-2 family of anti-apoptotic proteins navitoclax [18], was followed by many other new, and still less studied senolytic compounds [19–21]. In general, senolytics have been used in mouse models to alleviate senescence-related diseases [16, 22–24]

The use of senolytic compounds has been previously reported for the treatment of type 2 diabetes in mouse models. In particular, the combination of dasatinib and quercetin was used to alleviate metabolic dysfunction in diet-induced obese mice, attaining a reduction in the senescence markers SA-βGal and *Cdkn2a-p16*, and improved glucose and insulin tolerance tests [25]. Also, treatment with the senolytic navitoclax improved the β-cell function and fasting blood glucose levels in mice rendered diabetic by the insulin receptor antagonist S961, although navitoclax efficacy by glucose and insulin tolerance tests in a diet-induced obesity mouse model was not tested [5].

In our study, we compare in parallel the efficacy of navitoclax and the combination of D/Q in a mouse model of diet-induced obesity. We measure both their senolytic efficacy in white adipose tissue and their beneficial effects in glucose homeostasis. Also, we follow the metabolic phenotyping of our models with time, thus finely determining the time point at which each senolytic treatment is most efficacious. Our results showed that treatment with both navitoclax and D/Q improved insulin sensitivity after three cycles and glucose tolerance after four cycles, but these beneficial effects disappeared after 16 weeks of treatment.

## RESULTS

### Senolytic treatment strategy of obese and glucose intolerant mice

**Figure 1:**
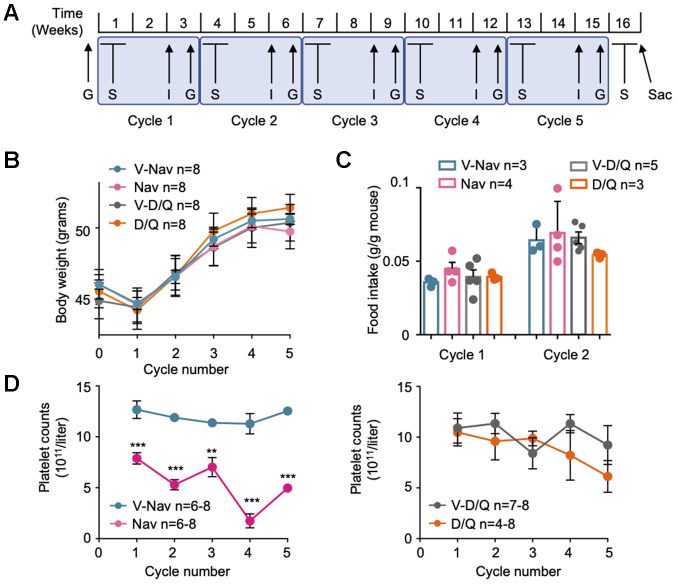
**Senolytic treatment strategy.** (**A**) Scheme of the senolytic treatments strategy: C57BL/6HsdOla male mice of 12 weeks of age were fed on 45% HFD for 4 months. Then, 5 cycles of senolytic treatment and glucose homeostasis assessment were undertaken: first, mice were treated daily by oral gavage with the senolytic or its vehicle for 5 consecutive days, and rested for 1 week. The third week of each cycle, an insulin tolerance test (I) and a glucose tolerance test (G) were performed. After the fifth cycle was finished, mice were treated again with senolytics for 5 consecutive days by oral gavage, and sacrificed at the fifth day of treatment (Sac). (**B**) Mouse body weight at the first day of every senolytic treatment, indicated for each of the 5 cycles. (**C**) Food intake recorded at cycles 1 and 2. (**D**) Blood samples were collected every fifth day of each cycle of senolytic treatments, right after the last oral administration, and platelet counts were determined. V-Nav: vehicle for navitoclax. Nav: navitoclax. V-D/Q: vehicle for dasatinib/quercetin. D/Q: dasatinib/quercetin. Bars and dots represent the average of the indicated number of mice. Error bars represent the standard error of the mean (s.e.m.). Statistical significance was assessed using the two-way ANOVA test with Sidak’s correction for multiple comparisons (**B**); the one-way ANOVA test with Tukey’s correction for multiple comparisons (**C**); or the unpaired two-tailed Student's t-test (**D**). **, p<0.01; ***, p<0.001.

To generate the mouse model of diet-induced obesity (DIO), we fed a cohort of 32 male mice of the C57BL/6OlaHsd background on a high-fat diet (HFD, 45% fat) for 4 months until they reached obesity. Then, we checked their glucose intolerance using a glucose tolerance test (GTT), and divided these mice in four groups of 8 mice. As shown in Supplementary Figure 1A and 1B, when we controlled for body weight gain or glucose intolerance at this point between the mice belonging to each experimental group, we observed that mice from all groups had evolved similarly until this timepoint. We then began a series of cycles of treatments with two of the best characterized senolytics: navitoclax and D/Q. More precisely, we performed 5 cycles of treatments, each cycle beginning with 5 consecutive days of administration of senolytics and their vehicles by oral gavage, followed by one week of rest, and a final week when we performed one insulin tolerance test (ITT) and one GTT. In total, each cycle lasted 3 weeks. For a detailed scheme, see Figure 1A. During all these cycles, mice were still fed with HFD. After an initial small weight loss during the adaptation to the treatments, that showed no difference between treatment groups, all mice gained weight constantly (Figure 1B). Also, we measured food intake during the first two cycles and did not observe any difference between treatment groups (Figure 1C). These results indicate that the senolytic treatments were not affecting the mouse body weight gain or food intake, thus proving the safety of these treatments in this metabolic scenario.

Finally, we verified the effectiveness of navitoclax treatment by following the blood platelet counts during all the cycles. As reported before for mice [26] and humans [27], blood platelet counts measured at the fifth day of navitoclax treatment by oral gavage were significantly decreased, compared with their corresponding control group and with the D/Q mixture and its control (Figure 1D), thus proving that navitoclax was effective in our model system.

### Efficacy of senolytic treatments in mouse models of obesity

**Figure 2:**
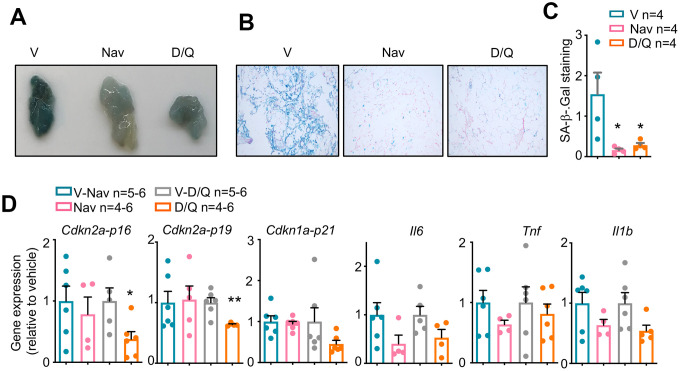
**Senescence markers in DIO-mice after senolytic treatment.** (**A**) Macroscopic view of perigonadal white adipose tissue (pWAT) from mice treated with the 5 cycles of senolytics and stained for senescence-associated β-Galactosidase (SA-β-Gal) activity. V: vehicle. Nav: navitoclax. D/Q:dasatinib/quercetin. (**B**) Microscopic images of the same pWATs shown in (**A**). (**C**) Quantification of the blue area, positive for SA-βGal, of 6 fields per condition from the pWATs shown in (**A**) and (**B**). (**D**) mRNA expression analysis of the indicated genes in pWAT obtained at the day of sacrifice. Statistical significance was assessed using the one-way ANOVA test with Tukey's correction for multiple comparisons (**C**) or the two-tailed unpaired Student's t-test between each treatment and its control (**D**) Asterisks refer to the comparison with the corresponding vehicle-treated mice. *, p<0.05; **, p<0.01.

After finishing the 5 cycles of treatment and metabolic phenotyping, we treated again with senolytics for 5 consecutive days by oral gavage and sacrificed right after the last treatment. We focused on perigonadal white adipose tissue (pWAT), because this tissue has been reported to present the most evident induction of senescence after obesity onset [3, 25]. We checked the efficacy of our senolytic treatments by measuring different senescence markers. First, we performed a whole-mount staining for SA-βGal, one of the best described senescence biomarkers [11]. As shown in Figures 2A–2C, after the last 5 days of treatment once all cycles had been finished, pWAT from vehicle-treated mice showed a strong staining for SA-βGal, while treatment with both navitoclax and D/Q significantly reduced the SA-βGal staining. We did not observe any significant changes in the weight of several tissues from these mice, indicating that the senolytic treatments did not induce apparent macroscopic tissue changes (Supplementary Figure 1C). We finally checked the mRNA expression of a number of senescence markers, including the tumor suppressors *Cdkn2a-p16*, *Cdkn2a-p19* and *Cdkn1a-p21*; and the senescence-associated cytokines *Il6*, *Tnf* and *Il1b.* We observed significant reductions in the pWAT expression of the senescence markers *Cdkn2a-p16* and *Cdkn2a-p19* after treatment with D/Q, but navitoclax-treated pWAT did not reduce the levels of any of these tumor suppressors. Also, we observed a clear but not significant tendency to reduced expression in the inflammation genes *Il6*, *Il1b* and *Tnf* for both treatments (Figure 2D).

### Glucose metabolism in mice treated with senolytics

**Figure 3:**
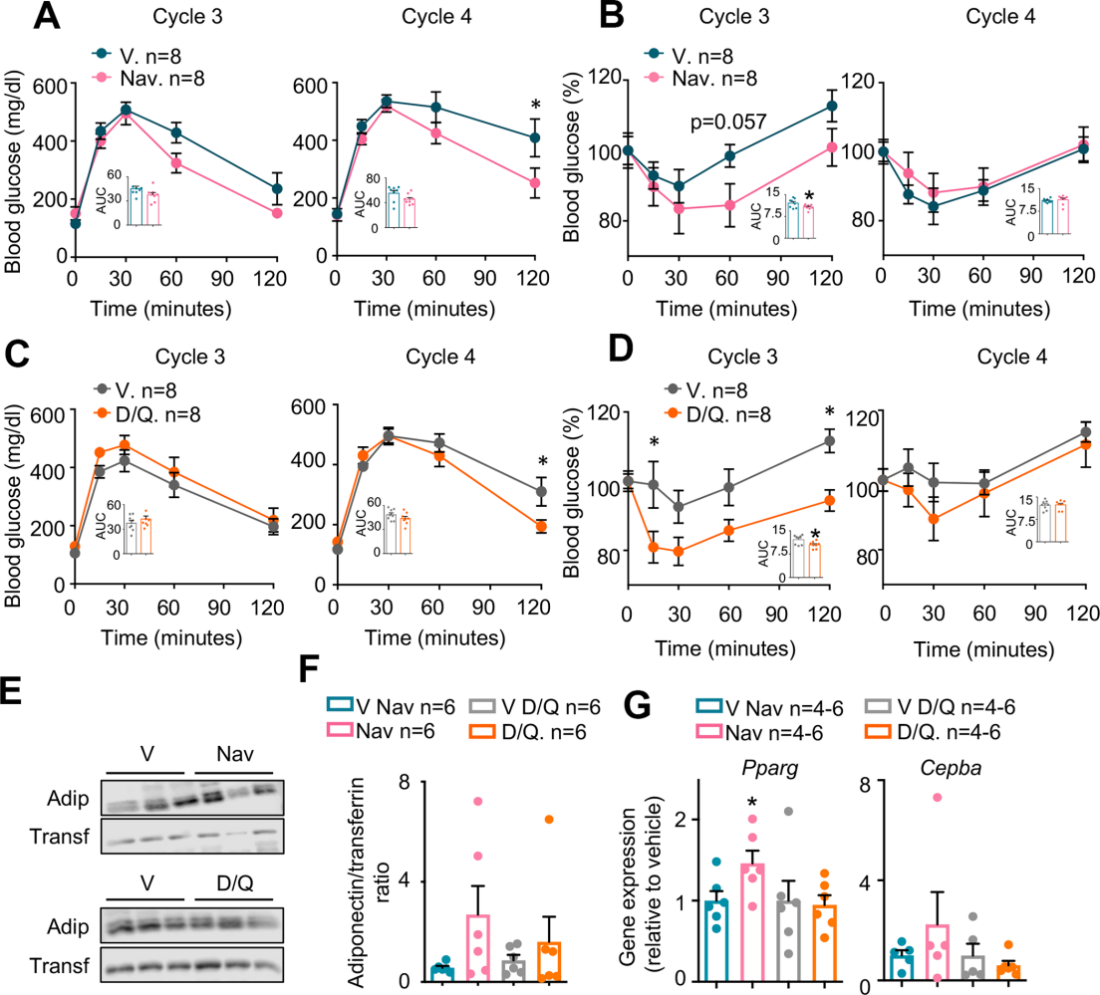
**Metabolic phenotyping of mice treated with senolytics.** Glucose (**A** and **C**) and insulin (**B** and **D**) tolerance tests performed at the indicated cycles. The area under the curve (AUC) was calculated for each experiment and represented in the insets. (**E**) Representative examples (n=3 from a total of n=6) of Western blots of the indicated proteins from plasma obtained at cycle 4 from the same mice shown in (**A**–**D**). (**F**) Quantification of Western blots for adiponectin, including those represented in (**E**), from all available plasma samples from mice shown in (**A**–**D**) (n=6). (**G**) mRNA expression analysis of the indicated genes in pWAT obtained at the day of sacrifice. Bars and dots represent the average of the indicated number of mice per group. Error bars represent the standard error of the mean. Statistical significance was assessed by the two-way ANOVA test with Sidak’s correction for multiple comparisons for the time course experiments (main graphs at **A**–**D**); and with the two-tailed unpaired Student's t-test for the AUC data (**A**–**D**) and for panels **E** and **F**. Asterisks refer to the comparison with the corresponding vehicle-treated mice. *, p<0.05.

As shown in Figure 2, both senolytic treatments had been effective in reducing senescence markers in the pWAT. To determine the metabolic effects of these treatments, we performed an insulin tolerance test (ITT) followed by a glucose tolerance test (GTT) during the third week of each cycle, after more than one week of rest from the senolytic treatments. Interestingly, GTTs were improved in mice treated with either navitoclax (Figure 3A) or D/Q (Figure 3C) when compared with their respective control-treated mice during cycle 4, but showed no difference in previous or following cycles (Supplementary Figure 1D and 1F). In turn, ITTs in mice treated with either navitoclax (Figure 3B) or D/Q (Figure 3D) also were improved during the cycle 3, preceding the GTT improvement; and were not affected in previous or following cycles (Supplementary Figure 1E, 1G). These results indicate that both senolytic treatments induced a transient improvement in glucose homeostasis after 3-4 cycles of treatments, that disappeared even if senolytic treatments were continued.

To further characterize the metabolic effects of navitoclax and D/Q in obese mice, we measured the plasma levels of the adipokine adiponectin, that has been extensively reported to be decreased in diabetic individuals both in mice [28] and in humans [29]. In particular, we measured plasma adiponectin at the last day of senolytic administration at the cycle 4 (where GTTs were most clearly improved, see Figure 3A and 3C). As shown in Figure 3E, 3F, there was a clear tendency in both senolytic treatments to increase plasma adiponectin, especially with navitoclax treatment, although these differences were not significant. Expression of the adipogenic genes *Pparg* and *Cebpa* at pWAT has been shown to correlate with insulin sensitivity [30, 31]. We thus measured the gene expression of *Pparg* and *Cebpa*, and observed no change in the D/Q-treated tissues, but an increased expression of these two markers in pWAT of navitoclax-treated mice, that reached significance in the case of *Pparg* (Figure 3G).

## DISCUSSION

Previous studies have reported that senescence plays an important role in obesity [32], obesity-induced anxiety and impaired neurogenesis [33] and in type 2 diabetes [3, 4]. As a consequence, removal of senescent cells either genetically (in the INK-ATTAC mouse model) or by senolytic treatments, has been proposed as an efficient therapy against senescence-related diseases [15, 33–35]. In 2019, two publications demonstrated that senescent cells removal by treatment with D/Q [25] or navitoclax [5] improved the glucose homeostasis in mouse models of type 2 diabetes.

In our work, we have studied the metabolic effect of the long-term treatment with navitoclax or D/Q in a diet-induced obesity mouse model. Although our experiments included only a limited amount of mice, our data clearly show that both senolytic treatments did not affect body weight (Figure 1B), food intake (Figure 1C), or tissue weight (Supplementary Figure 1C). In contrast, we observed the same body weight gain in both treatments, parallel to their respective vehicle controls, indicating that none of them prevented body weight gain with high fat diet (Figure 1B). Previous reports showed that HFD induced senescence most evidently in pWAT [25, 32, 36]. Confirming these findings, we observed a reduction in whole mount SA-βGal staining in pWAT after 5 cycles of both senolytic treatments (Figure 2A–2C), as previously described for D/Q in pWAT [25]. In addition, we showed for the first time that navitoclax also efficiently reduced SA-βGal activity in pWAT. We also analyzed pWAT mRNA expression of the senescence markers *Cdkn2a-p16*, *Cdkn2a-p19* and *Cdkn1a-p21*, and confirmed previous reports showing a reduced *Cdkn2a-p16* expression after D/Q treatment [25]. In addition, we also observed for the first time a significant reduction of the expression of *Cdkn2a-p19* in pWAT after D/Q treatment. In contrast and to our surprise, in the case of navitoclax we did not observe changes in these senescence markers. These results are in agreement with previous reports, where treatment with navitoclax in insulin insensitive mice did not elicit any change of *Cdkn2a-p16* nor *Cdkn1a-p21* in pWAT [5]. This indicates that these two senolytic treatments follow different molecular pathways. In addition, we observed that both senolytics induced a non-significant but evident reduction in the mRNA expression of the SASP factors *Il6*, *Tnf* and *Il1b*, proposed as mediators of insulin resistance [4, 37].

We then analyzed glucose and insulin sensitivity to measure the diabetic profile in our mice after every senolytic cycle. We observed a different behavior of between mice treated with each type of vehicle at the ITT experiments from cycle 3 (Figure 3B, 3D, Supplementary Figure 1E and 1G), probably due to the different composition of both vehicles (15% DMSO + 15% PEG 400 for navitoclax; and 10% PEG 400 for D/Q). In any case, the improved insulin sensitivity of the senolytic-treated mice observed in cycle 3 disappears in later cycles, independently of their vehicles. Therefore, according to our statistically significant results from GTT and ITTs, we conclude that there is a transient improvement in glucose homeostasis in senolytic-treated mice that peaks around cycles 3 and 4. In addition, our data coincide in intensity and time of appearance with previous reports with D/Q [5, 25] (Figure 3A–3D). Navitoclax treatment has already been shown to improve blood fasting glucose levels in a pharmacological type 2 diabetes model of treatment with the insulin receptor antagonist S961 [5]; but it had never been tested with a mouse model of DIO, as presented here. Also, at the fourth cycle we observed a tendency to increased blood adiponectin, an adipokine previously described as an enhancer of insulin sensitivity [38]. In addition, we observed in pWAT after the 5 treatment cycles an increased expression of the adipocyte markers *Pparg* and *Cebpa,* both crucial for a proper adipocyte differentiation and insulin sensitivity [30, 31, 39, 40]. These beneficial effects were more evident in the navitoclax-treated mice, although they only reached significance in the case of *Pparg* (Figure 3G).

In addition, our results also extend the glucose metabolism studies to 5 cycles of treatment, spanning 16 weeks, and demonstrate that GTT and ITT improvements are only apparent during a short period of time, and are lost in subsequent treatments (Supplementary Figure 1D–1G). These data suggest that senolytic chronic treatments exert a transient effect in glucose sensitivity, that peaks after several weeks of treatment but diminishes with time. From our data we can only speculate about the reasons of this transient effect. First, the doses of senolytics can be optimized to increase their beneficial effects on glucose intolerance, although the thrombocytopenia elicited by navitoclax (Figure 1D) strongly limits the maximum dose. Also, senolytics were administered daily for 5 days, and the ITT and GTT were only performed 10 days after the last oral gavage. This delay, intended to allow for recovery from the senolytic treatments, can also have obscured the physiological effects of these treatments. Finally, from a clinical perspective, the use of senolytics to treat insulin insensitivity should be accompanied by nutritional interventions reducing fat and carbohydrates intake. Instead, mice shown here were kept on high-fat diet throughout all the treatment with senolytics. We hypothesize that this nutrient overload, when kept in time, can reduce the efficacy of the treatments with senolytics. Future research should discern whether the combination of senolytics with nutritional interventions, which would mimic more closely the intended clinical scenario, is more effective against diabetes.

Altogether, our experiments comparing navitoclax and D/Q in obese, diabetic mice show that both treatments are effective in improving several metabolic markers, as glucose and insulin tolerance tests, adipogenic genes or blood adiponectin. However, these beneficial effects are only apparent during a short period of time and disappear later, at least for glucose and insulin tolerance. These results clearly show that senolytic administration is a promising strategy for the treatment of obesity and diabetes. In our case, we have established very restrictive treatment conditions, maintaining the high-fat diet throughout all the senolytic treatment, and treating only for 5 days every three weeks. Based on our work, we hypothesize that treatment with different drug doses and/or increased frequency of senolytic treatments, together with diet management, could constitute a potent and safe strategy for these conditions in mice and humans.

## MATERIALS AND METHODS

### Animal experimentation

Animal experimentation at the CNIO, Madrid, was performed according to protocols approved by the CNIO-ISCIII Ethics Committee for Research and Animal Welfare (CEIyBA number CBAO6_2018).

### Diet-induced obesity

A cohort of 32 male mice of C57BL/6HsdOla background of 12 weeks of age were induced to develop diet-induced obesity (DIO) by feeding them during 4 months on irradiated 45% high fat diet (HFD, Research Diets, D12451). During this time, body weight was recorded every two weeks. After these 4 months on HFD, a glucose tolerance test was performed to confirm glucose intolerance.

### Treatment cycles: senolytic treatments and metabolic assays

Obese, glucose intolerant mice were divided in cohorts of 8 animals with the same body weight average between groups, and housed in cages of 1-3 mice per cage (4 cages for navitoclax animals; 3 cages for navitoclax vehicle; 3 cages for D/Q animals; and 5 cages for D/Q vehicle). At the beginning of each cycle, mice were weighed (this body weight record is shown at Figure 1B) and treated by oral gavage with either navitoclax (MedChemExpress, HY-10087; 50 mg/kg dissolved in 15% DMSO and 15% PEG 400); with a mixture of dasatinib (MedChemExpress, HY-10181; 5 mg/kg dissolved in 10% PEG 400), and quercetin (MedChemExpress, HY-18085; 50 mg/kg dissolved in 10% PEG 400); or with their corresponding vehicles (15% DMSO and 15% PEG 400 for navitoclax, or 10% PEG 400 for D/Q). A total of 5 cycles of treatment were performed. In each cycle, senolytic treatment was given for 5 consecutive days, followed by two weeks of no senolytic treatment, during which metabolic experiments were performed.

Food intake, recorded during the first two cycles of treatment, is expressed as the average of the food eaten by each mouse in a given treatment, calculated as the total food eaten in each cage, divided by the number of mice in that cage. all the cages of a given treatment

For glucose metabolism assays, on the first day of the third week of each cycle, an insulin tolerance test (ITT) was performed by intraperitoneal injection of 0.75 U/kg insulin dissolved in saline (Insulin Humulina 100 U/ml, Lilly). 2 days later a glucose tolerance test (GTT) was performed: mice were fasted from 17:00 hours until the next day at 10:00 hours (17 hours of fasting), and then injected intraperitoneally with 2 g/kg dextrose (Sigma, G7021) dissolved in water. For both ITT and GTT, blood glucose levels were measured from an incision in the tail 0, 15, 30, 60 and 120 minutes after insulin or glucose injection using a portable glucometer (Menarini Diagnostics).

For hemograms, blood samples were collected from each mouse at each cycle by submandibular bleeding in EDTA-coated tubes after the last senolytic oral gavage. Platelet numbers (PLT 10^11^/liter) were measured in a Laser Cell blood counter (CVM veterinary diagnostic).

### Laboratory experimentation

### Senescence-associated β-galactosidase staining

We modified a senescence-associated β-galactosidase (SA-βGal) staining protocol from Nature protocols [41]. Briefly, frozen pWAT was sectioned and then fixed at room temperature for 1 hour in 2% formaldehyde / 0.2% glutaraldehyde, washed twice with washing solution (PBS, 2 mM MgCl_2_ pH 6.0), and incubated for 6 hours at 37ºC with SA-βGal solution (20 mg/ml X-Gal in dimethylformamide; 100 mM K4[Fe(CN)6]; 100 mM K3[Fe(CN)6]; 150 mM NaCl in PBS; 2 mM MgCl_2_ pH 6.0). All these components were purchased from Sigma. After this incubation, macroscopic images were taken to determine differences between vehicle and treatment. Incubation in SA-βGal solution was then continued for 20 extra hours, and then tissues were washed twice with PBS and dehydrated with increasing EtOH concentrations (30% for 15 minutes, and then 70% for 30 minutes). Samples were then kept in 70% EtOH until embedded in paraffin, sectioned and counterstained with nuclear fast red for microscope image acquisition. Analysis of SA-βGal staining were performed from 6 fields per animal using the ImageJ software.

### Quantitative real-time PCR

RNA from pWAT tissue was isolated using TRI Reagent (MRC, TR-118) according to manufacturer’s instructions. After nanodrop quantification, 1 μg of total RNA was retrotranscribed into cDNA using High Capacity cDNA reverse transcription kit (Applied biosystems, 4368814). Quantitative real-time PCR was performed using SYBR green PCR master mix (Promega, A6002). Relative RNA expression was normalized using the *Actb* and *36b4* housekeeping genes.

Primer sequences are: *Mm-β-actin* Fwd: GGACCACACCTTCTACAATG; Rvs: GTGGTGGTGAAGCTGTAGCC; *Mm-36b4* Fwd: AGATTCGGGATATGCTGTTGG; Rvs: AAAGCCTGGAAGAAGGAGGTC; *Mm-Cdkn2a-p16* Fwd: TACCCCGATTCAGGTGAT; Rvs: TTGAGCAGAAGAGCTGCTACGT; *Mm- Cdkn2a-p19* Fwd: GCCGCACCGGAATCCT; Rvs: TTGAGCAGAAGAGCTGCTACGT; *Mm-Cdkn1a-p21* Fwd: TCCACAGCGATATCCAGACA; Rvs: GGACATCACCAGGATTGGAC; *Mm-Il6* Fwd: GTTCTCTGGGAAATCGTGGA; Rvs: GGTACTCCAGAAGACCAGAGGA; *Mm-Tnf* Fwd: GCCTCTTCTCATTCCTGCTT; Rvs: CTCCTCCACTTGGTGGTTTG; *Mm-Il1b* Fwd: AAAAGCCTCGTGCTGTCG; Rvs: AGGCCACAGGTATTTTGTCG; *Mm-Cebpa* Fwd: CCGGGAGAACTCTAACTC; Rvs: GATGTAGGCGCTGATGT; *Mm-Pparg* Fwd: AGGCCGAGAAGGAGAAGCTGTTG; Rvs: TGGCCACCTCTTTGCTCTGCTC.

### Measurement of blood adiponectin and transferrin

Blood samples obtained from submandibular bleeding on the last day of senolytic administration at cycle 4 were collected in EDTA tubes. Plasma was obtained after centrifugation at 4000xg for 5 minutes. Protein extracts were prepared diluting the blood 1:10 in lysis buffer (50 mM Tris pH 7.4; 150 mM NaCl; 0.5% NP-40; 1mM phenyl-methyl-sulfonyl fluoride; protease inhibitors cocktail (Sigma-Aldrich P8340); 1mM NaF; and 10mM sodium orthovanadate).

We performed sodium dodecyl sulfate polyacrylamide gel electrophoresis (SDS-PAGE) of 10 μg of total protein extracts using 10% polyacrylamide mini-gels. The proteins were transferred to nitrocellulose membranes and incubated for one hour at RT in blocking buffer (5% skimmed milk in Tris-buffered saline (TBS); 0.1% Tween-20 (TBS-T)). The primary antibody was hybridized overnight at 4ºC in 5% BSA, TBS-T using antibodies against adiponectin (1:1000, Abcam, ab22554) or transferrin (1:1000, Santa Cruz, sc373785). Then, membranes were washed three times in TBS-T and incubated in 5% skimmed milk with a secondary fluorescence anti-mouse antibody (1:20.000, LICOR, goat anti-mouse c50113). Band quantification was performed in an Odyssey Fc detector. Band intensity was quantified using the ImageJ software, and the adiponectin/transferrin ratio was calculated.

### Statistical analysis

Statistical analyses were performed using GraphPad Prism 6.01 for Windows. Precise statistical tests are specified at each figure legend. Data was collected for as many samples/mice as possible, although technical limitations reduced the number of replicates in some assays. Statistical outliers were identified using the ROUT method with Q=1% (GraphPad). Differences between two groups were analyzed using the two tailed Student’s t-test. Differences between three or more groups were analyzed using One-way ANOVA with Tukey’s correction for multiple comparisons. Differences between three or more groups defined in more than one variable were analyzed using Two-way ANOVA with Sidak´s correction for multiple comparisons. All analyses were performed as paired or unpaired tests, depending on the type of analyzed samples.

## Supplementary Material

Supplementary Figure 1
