# Supplementary material for: Transient metabolic improvement in obese mice treated with navitoclax or dasatinib/quercetin
Source: Aging (Albany NY). 2020 Jun 25;12(12):11337–48. doi: 10.18632/aging.103607 (PMC7343475; doi:10.18632/aging.103607)
Supplement: Supplementary Figure 1 [file aging-12-103607-s001..pdf]

SUPPLEMENTARY FIGURE

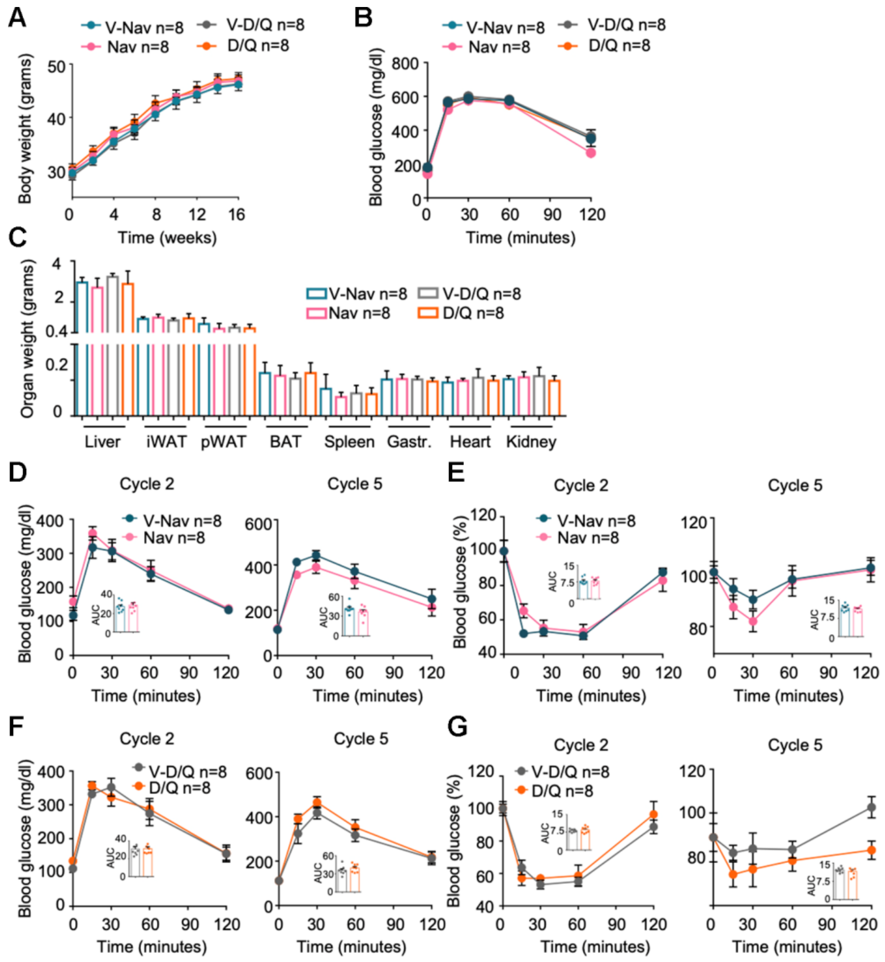

Supplementary Figure 1. Metabolic phenotyping of mice treated with senolytics. (**A**) Mouse body weight during the 16 weeks of high-fat diet treatment. (**B**) GTT in obese mice after the 16 weeks of high-fat diet treatment. In (**A** and **B**) all mice were treated as a single cohort of 32 mice. We represent the mice separated in the 4 different experimental groups to show that there was no difference in body weight or glucose tolerance between experimental groups before treatments with senolytics began. (**C**) Weights of the indicated tissues at the time of sacrifice ("Sac" in Figure 1A). iWAT: inguinal white adipose tissue. pWAT: perigonadal white adipose tissue. BAT: brown adipose tissue Gastr: gastrocnemius. (**D**–**G**) GTTs (D and F) and ITTs (**E** and **G**) at the indicated cycles and with the indicated treatments of the same mice used in Figure 3. The area under the curve (AUC) is shown as an inset in each panel. Bars and dots represent the average of the indicated number of mice per group. Error bars represent the standard error of the mean. Statistical significance was assessed by the two-way ANOVA with Sidak's correction for multiple comparisons for the time course experiments (**A**, **B** and main graphs at **D**–**G**); the one way ANOVA test with Tukey's correction for multiple comparisons (**C**); and with he two-tailed unpaired Student's t-test for the AUC data (**D**–**G**).
